# Episodic cluster headache with suspected giant cell arteritis, sinusitis-related headache, and dietary triggers: a case report

**DOI:** 10.3389/fpain.2026.1884385

**Published:** 2026-07-23

**Authors:** Shusheng Jiao, Ning Zhen

**Affiliations:** 1 Department of Neurology, Bethune International Peace Hospital, Shijiazhuang, Hebei, China; 2 The Fifth Dispensed Outpatient Department, Bethune International Peace Hospital, Shijiazhuang, Hebei, China

**Keywords:** cluster headache, giant cell arteritis, sinusitis-related headache, soy sauce, vinegar

## Abstract

**Background:**

Cluster headache is a primary trigeminal autonomic cephalalgia characterized by severe, strictly unilateral orbital/periorbital pain with ipsilateral autonomic features. It is rare for a patient with cluster headache to have coexisting multiple headache disorders and experience unusual triggers.

**Case presentation:**

A 53-year-old male with a 14-year history of episodic cluster headache presented with his third cluster episode. He exhibited strictly unilateral left orbital/periorbital/temporal drilling pain [Visual Analogue Scale (VAS) 9–10/10], ipsilateral conjunctival injection, lacrimation, rhinorrhea, and agitation. Notably, during the second episode (2015), left maxillary sinusitis was identified and surgically treated, leading to immediate and complete headache resolution. During the current episode, physical examination revealed left temporal artery nodularity and tenderness—raising suspicion of giant cell arteritis. However, the erythrocyte sedimentation rate and C-reactive protein levels were within the normal range. Brain MRI/MRA revealed only nonspecific white matter changes, along with suspected wall thickening of the left superficial temporal artery. The patient declined temporal artery ultrasound, high-resolution MRI, and biopsy. In accordance with clinical guidelines, the patient was administered comprehensive treatment for cluster headache. Given the suspicion of giant cell arteritis, empirical prednisone was also initiated at a dose of 1 mg/kg/day. By day 3 of treatment, attack frequency had reduced from daily to 3–4 times per week, pain intensity decreased from VAS 10/10 to 3–6/10, attacks resolved within 30 min either spontaneously or after zolmitriptan, and temporal artery nodules had markedly diminished. Interestingly, during follow-up, the patient reported that ingestion of soy sauce or vinegar consistently triggered an unusual throat discomfort followed by headache within 30 min, whereas avoidance of these substances resulted in no attacks. The patient's strict avoidance of soy sauce and vinegar led to a marked reduction in headache frequency over a short period, ultimately resulting in a complete absence of headache episodes. He remains under follow-up.

**Conclusion:**

This case highlights an unusual concurrence of episodic cluster headache with suspected giant cell arteritis and possible sinusitis-related headache in a single patient. The potential role of soy sauce and vinegar as triggers for cluster headache warrants further validation with extended follow-up and a larger case series.

## 1 Introduction

Cluster headache (CH) is a debilitating primary trigeminal autonomic cephalalgia characterized by severe, strictly unilateral orbital, supraorbital, temporal, or combined pain, accompanied by ipsilateral autonomic features such as conjunctival injection, lacrimation, rhinorrhea, and restlessness or agitation (1). Although the pathophysiology of CH remains incompletely understood, the hypothalamus is considered a key player in its circadian and circannual periodicity, and the trigeminovascular system mediates the nociceptive signals (2). Notwithstanding that the diagnosis of CH is primarily clinical, in accordance with the International Classification of Headache Disorders, 3rd edition (ICHD-3) criteria (3), its clinical picture is liable to be confounded by overlapping features with other headache disorders, rendering it susceptible to substantial diagnostic delay or misclassification (4).

In clinical practice, several conditions can mimic or coexist with CH, leading to diagnostic challenges. Giant cell arteritis (GCA), an immune-mediated vasculitis affecting medium and large arteries, commonly presents with new-onset headache, scalp tenderness, and jaw claudication in individuals aged ≥50 years (5). Overlap between CH and GCA is rarely reported (6, 7), but distinguishing the two is critical because GCA requires prompt glucocorticoid therapy to prevent irreversible vision loss (8). Sinusitis-related headache is another potential confounder (9). Although acute sinusitis can cause unilateral facial pain, true sinus headache is overdiagnosed, and up to 88% of self-diagnosed “sinus-related headache” cases actually meet criteria for migraine or other primary headache disorders (10, 11). Nevertheless, sinusitis may occasionally coexist with CH or even trigger CH attacks in susceptible individuals (12).

Beyond diagnostic mimics, dietary triggers of CH are less well characterized compared with migraine. Alcohol is the only well-established and most potent dietary trigger for CH (13, 14), provoking attacks within 1 h in up to 50%–80% of patients during an active cluster period (15). Other potential triggers such as histamine, tyramine, monosodium glutamate, and nitrates have been implicated in headache disorders, but their specific role in CH remains unclear (16). Fermented condiments, including soy sauce and vinegar, contain multiple biogenic amines and vasoactive substances; however, their potential to trigger CH has not been systematically reported.

We describe a 53-year-old male with a 14-year history of episodic CH whose third episode was complicated by suspected GCA and prior sinusitis-related headache. Most notably, the patient consistently reported that ingestion of soy sauce or vinegar triggered CH attacks within 30 min, with complete remission upon avoidance. This case highlights the diagnostic complexity of CH in middle-aged patients, the challenges of distinguishing CH from GCA and sinusitis-related headache, and the potential role of fermented condiments as novel dietary triggers for CH.

## 2 Case presentation

A 53-year-old male with a 14-year history of episodic headache presented to our neurology department in April 2026 with a seven-day history of severe left-sided headache. The patient has experienced a total of three distinct CH episodes over 14 years. The study was approved by the Institutional Review Board of Bethune International Peace Hospital, and written informed consent was obtained from the patient for publication of this case report and any accompanying images.

### 2.1 First episode

(April 2012): At age 39, the patient experienced a sudden onset of severe left orbital, periorbital, and temporal pain. The attacks occurred once daily, consistently around 10:00 AM, lasting 30 min to 3 h. The pain was strictly unilateral, characterized by a drilling sensation in the left orbit and periorbital region, accompanied by a pulsating component in the left temporal region. During attacks, the patient exhibited agitation (inability to lie still, pacing, restlessness, rocking his body). The pain intensity was rated 9/10 on the Visual Analog Scale (VAS). Ipsilateral autonomic features included conjunctival injection (red eye), lacrimation, rhinorrhea, and eyelid edema. No ptosis, visual blurring (miosis), or abnormal forehead/facial sweating was reported. The patient denied nausea, vomiting, photophobia, or phonophobia during or between attacks. No clear triggers were identified. At a local hospital, diagnosis details were unavailable, and no imaging or laboratory tests were performed. Treatment consisted of lidocaine-soaked cotton balls placed intranasally during attacks, which relieved pain within 20 min. The episode resolved spontaneously after 10 days.

### 2.2 Second episode

(March 2015): At age 42, the patient again experienced sudden-onset left orbital, periorbital, and temporal headaches. The pain quality, location, pattern, autonomic symptoms, and attack frequency were nearly identical to the first episode, but with greater severity. He described it as “the most unbearable pain of my life” (VAS 10/10), and exhibited head-banging and fist-pounding behaviors in an attempt to relieve the pain. Attacks occurred once daily (predominantly at 10:00 AM, occasionally at 3:00 PM), lasting >30 min but <3 h. Oral diclofenac sodium (50 mg) provided gradual relief. On day 6, he presented to our hospital's Otorhinolaryngology department. Nasal endoscopy and sinus CT revealed left maxillary sinusitis and left middle turbinate hypertrophy. A diagnosis of sinusitis-related headache was made by otorhinolaryngologist, and left maxillary sinus open surgery was performed. Notably, headache attacks ceased completely the day after surgery.

### 2.3 Third episode

**Figure 1:**
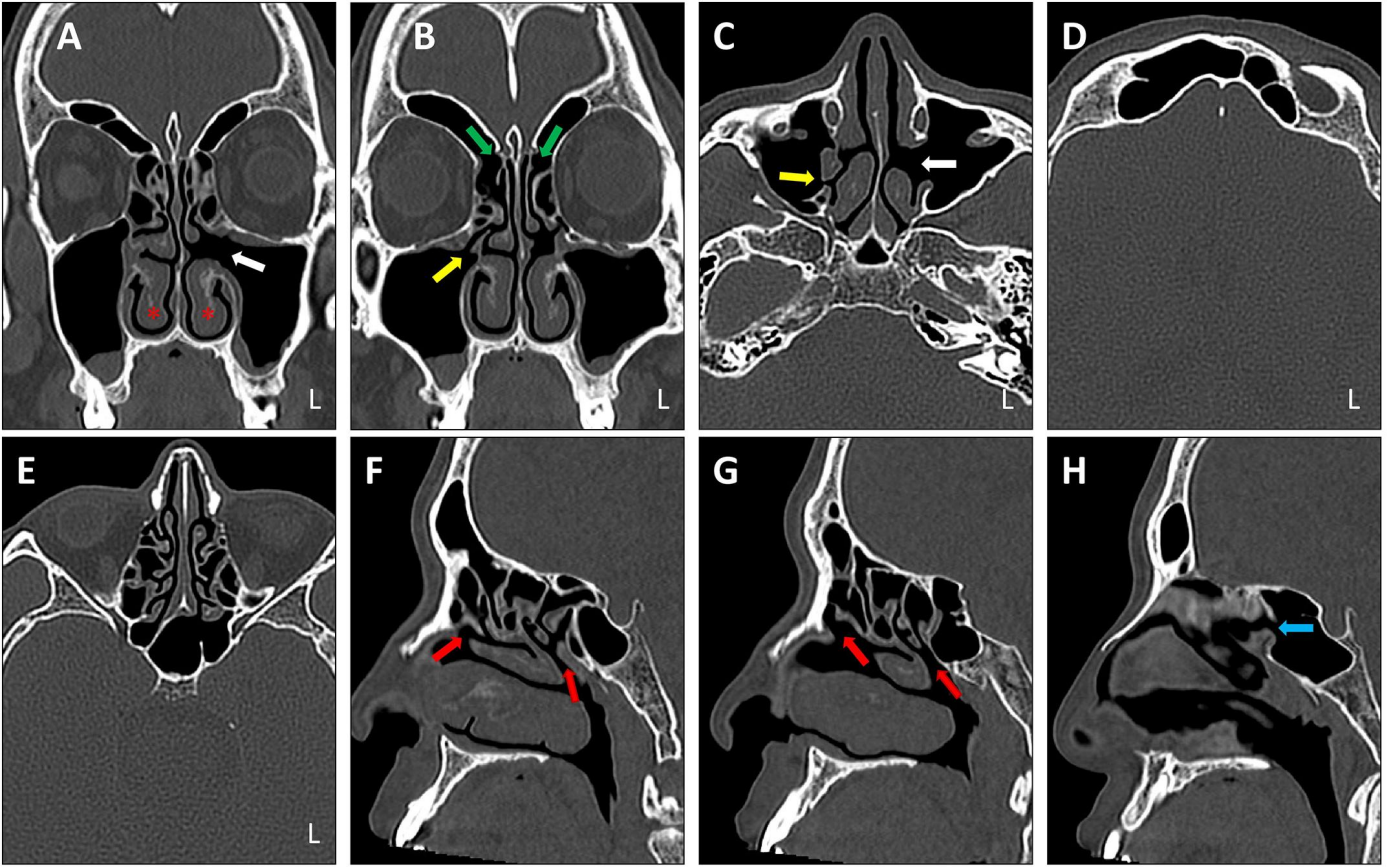
Sinus CT findings of the patient. The key link between headache and sinusitis lies in whether the drainage pathway is obstructed. In this patient, the sinus CT showed no definitive evidence of impaired drainage; therefore, the imaging findings do not support a confirmed diagnosis of sinusitis-related headache. **(A–D)** Postoperative changes are observed in the left maxillary sinus, with a widened ostium (white arrows in **A**, **C**); marked thickening of the sinus mucosa was observed on the medial, superior, and inferior walls of the left maxillary sinus, with partial nodular appearance; a small amount of fluid level was noted in the right maxillary sinus, with unobstructed ostium (yellow arrows in **B**, **C**); bilateral inferior turbinate hypertrophy was present (red asterisk in **A**), and the nasal cavity was basically unobstructed; bilateral frontal sinuses developed well **(D)**, without fluid level in the sinuses; no mucosal hypertrophy, mucus retention, cysts, polyps were found in the frontal sinus ostium/frontal recess area, and the ostium was unobstructed (green arrow in **B**). **(E–G)** Bilateral ethmoid air cells developed well with intact bony septa, and mild thickening of the bilateral ethmoid sinus mucosa was seen **(E)**; the openings of the anterior and posterior ethmoid air cells were unobstructed (red arrows: F for left ethmoid sinus, G for right). **(H)** The sphenoid sinus was well pneumatized without mucosal thickening; the sphenoid ostium was unobstructed, and no soft tissue obstruction was observed (blue arrow).

(April 1, 2026): At age 53, the patient experienced a recurrence of left orbital, periorbital, and temporal headache (VAS 10/10) with ipsilateral conjunctival injection, lacrimation, and rhinorrhea. The pain characteristics, pattern, autonomic symptoms, attack frequency, severity, and behavioral manifestations were identical to the second episode. He first sought care at a community clinic, where oral diclofenac sodium, acupuncture, and hot compress therapy provided relief within 30–45 min, though episodes still occurred daily. On day 7, he returned to our otorhinolaryngology department. Nasopharyngeal CT showed postoperative changes, paranasal sinusitis, bilateral nasal mucosal thickening, bilateral middle/inferior turbinate hypertrophy, and mild nasal septum deviation (Figure 1). Nasal endoscopy revealed: history of nasal septum correction and left maxillary sinus open surgery; the left maxillary sinus ostium was patent; bilateral nasal mucosa showed chronic congestion with bilateral inferior turbinate hypertrophy; no visible neoplasms or purulent secretions; the nasopharyngeal mucosa was smooth without visible neoplasms. The otorhinolaryngologist considered that the current sinus condition could not explain the patient's headache symptoms and recommended an ophthalmology consultation. Ophthalmologic examination (fundoscopy and intraocular pressure) was unremarkable, prompting referral to Neurology. At the time of the neurology visit, the patient was headache-free, and neurological examination revealed no positive findings. He was admitted to our department for further evaluation and management under the working diagnosis of cluster headache.

**Figure 2:**
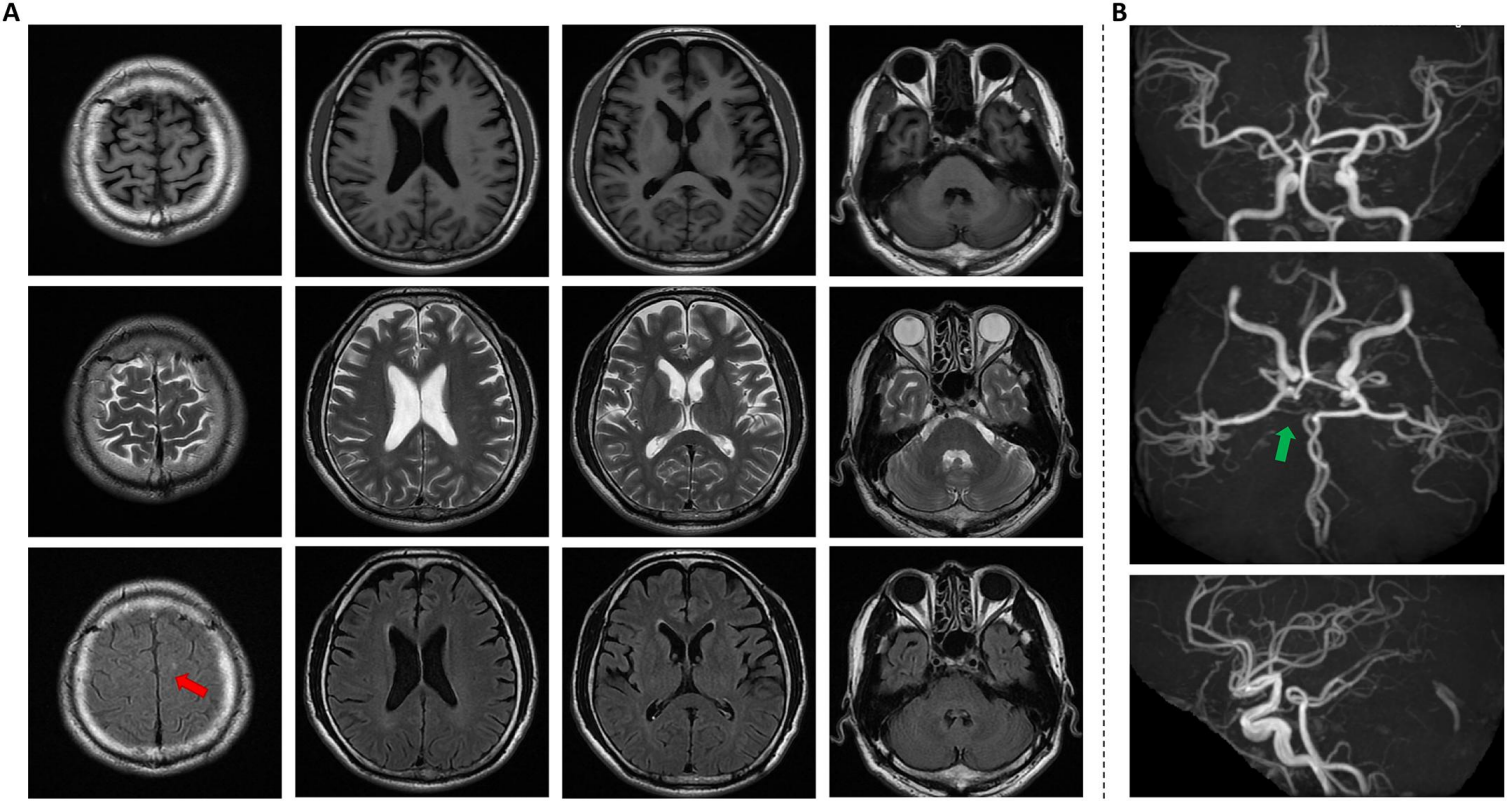
Cranial MRI + MRA findings of the patient. **(A)** Axial images of 4 representative slices (upper: T1-weighted, middle: T2-weighted, lower: T2-FLAIR), which revealed punctate white matter degeneration in the left frontal lobe (red arrow). **(B)** Multi-angle MRA indicated that the A1 segment of the right anterior cerebral artery was slender, suggestive of a developmental variant (green arrow). No imaging features of common etiologies of secondary cluster headache were observed, including pituitary adenoma, cerebral arteriovenous malformation, internal carotid artery aneurysm, and cerebral venous sinus thrombosis.

**Figure 3:**
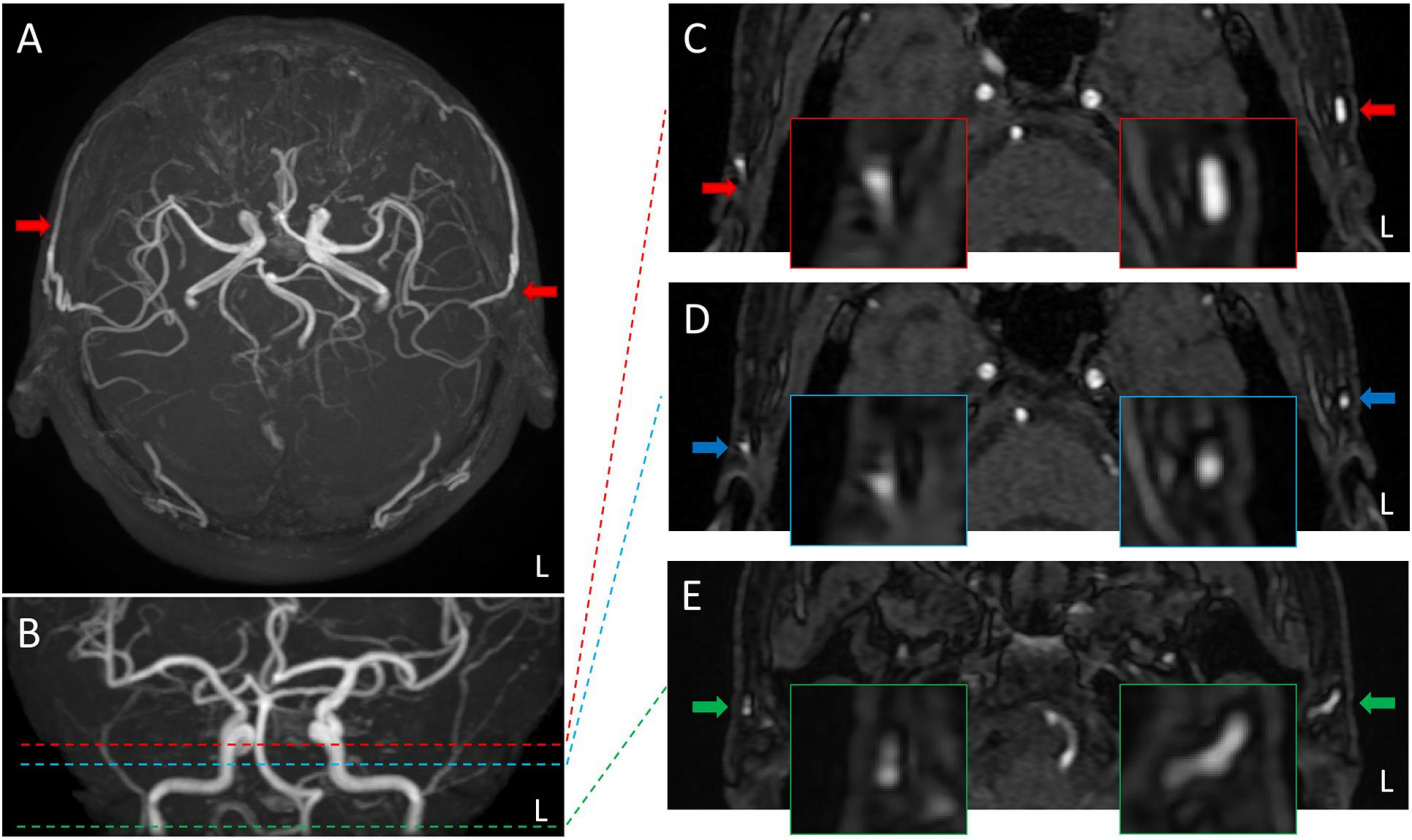
Figure 3. 3D-TOF source axial images suggesting possible giant cell arteritis. **(A)** 3D-TOF images show no obvious stenosis, dilation, or beaded changes in the bilateral superficial temporal arteries. **(B–E)** Careful review of the axial source images at multiple levels reveals that the wall of the left superficial temporal artery is significantly thickened compared with the contralateral side (**C**-**E**, arrows indicate the superficial temporal artery; insets show magnified views of the ipsilateral superficial temporal artery. Panel **B** indicates the corresponding levels of three representative axial slices).

Upon admission, a detailed history was obtained. The headaches had a sudden onset without aura or prodromal symptoms. During and around the attacks, there was no nausea, vomiting, photophobia, phonophobia, exacerbation with physical activity, masticatory or speech-related muscle fatigue or soreness, morning stiffness in the shoulders or neck, sudden vision loss or visual abnormalities, visual aura, limb weakness, or sensory disturbances. The patient maintained a strictly controlled diet, never consuming monosodium glutamate, and avoided foods containing scallions, ginger, garlic, chili, cheese, or cured meats. He had no history of alcohol or tobacco use and did not consume coffee. He reported a past history of hepatitis C, which had been cured, and had no chronic diseases, allergies, or infectious diseases. Interictal physical examination was unremarkable. During an attack, physical examination revealed thickening, nodularity, and marked tenderness of the left superficial temporal artery compared with the right; left conjunctival injection, lacrimation, and rhinorrhea were also observed. Based on the characteristics and location of the headache (strictly unilateral, left orbital, periorbital, or temporal boring pain, severe), attack pattern (clustered, periodic), and associated autonomic symptoms (conjunctival injection, lacrimation, rhinorrhea), a diagnosis of episodic cluster headache was made according to the ICHD-3 diagnostic criteria. Additionally, considering the patient's age (over 50 years), the acute onset of headaches over the past 10 years, scalp tenderness, and the superficial temporal artery findings, GCA (temporal arteritis) was also considered according to the 2022 ACR/EULAR (American College of Rheumatology/ European Alliance of Associations for Rheumatology) criteria. To exclude common etiologies of secondary cluster headache, including pituitary adenoma, cerebral arteriovenous malformation, internal carotid artery aneurysm, and cerebral venous sinus thrombosis, brain MRI combined with MRA and MRV was recommended. For further evaluation of GCA, erythrocyte sedimentation rate (ESR) and C-reactive protein (CRP) levels were measured. Unfortunately, the patient declined ultrasound, high-resolution MRI, and biopsy of the superficial temporal artery, along with cerebral MRV, due to financial constraints. Routine laboratory tests, including blood glucose, lipid profile, liver function, renal function, electrolytes, complete blood count, urinalysis, and stool analysis, were conducted. Results showed: Brain MRI + MRA revealed punctate white matter degeneration in the left frontal lobe; the A1 segment of the right anterior cerebral artery was slender, suggestive of a developmental variant (Figure 2). In consultation with two experienced radiologists, sequential review of MRI T2 and FLAIR sequences showed no evidence of cerebral venous sinus thrombosis. Notably, upon reviewing the source axial images of the 3D-TOF sequence and carefully comparing the cross-sectional appearances of the bilateral superficial temporal arteries, we found that the wall of the left superficial temporal artery appeared significantly thickened compared to the right side (Figure 3). No significant abnormalities were found in the intracranial venous system ESR was 2 mm/hour, and CRP was within the normal range. No other remarkable abnormalities were noted on laboratory testing.

Treatment was guided by the Chinese Guidelines for the Diagnosis and Treatment of Cluster Headache. Acute attacks were managed with high-flow oxygen inhalation and zolmitriptan nasal spray. In the interictal period, non-invasive vagus nerve stimulation (bilateral, once daily) and greater occipital nerve block (ipsilateral, three consecutive sessions) were administered. To address the possible coexistence of GCA, and in accordance with the Chinese Guidelines for the Diagnosis and Treatment of Cluster Headache, the patient was treated with oral prednisone at a dose of 1 mg/kg/day for 5 days, followed by a gradual taper of 5 mg every 5 days. After three days of treatment, headache attack frequency decreased to 3–4 times per week, with improvements in both pain intensity (VAS 3–6) and attack duration (most attacks resolved within 30 min, either spontaneously or following triptan use). The nodularity of the superficial temporal artery was markedly diminished and local tenderness resolved significantly, comparable to the contralateral side. The patient was discharged after two weeks and scheduled for regular follow-up.

**Figure 4:**
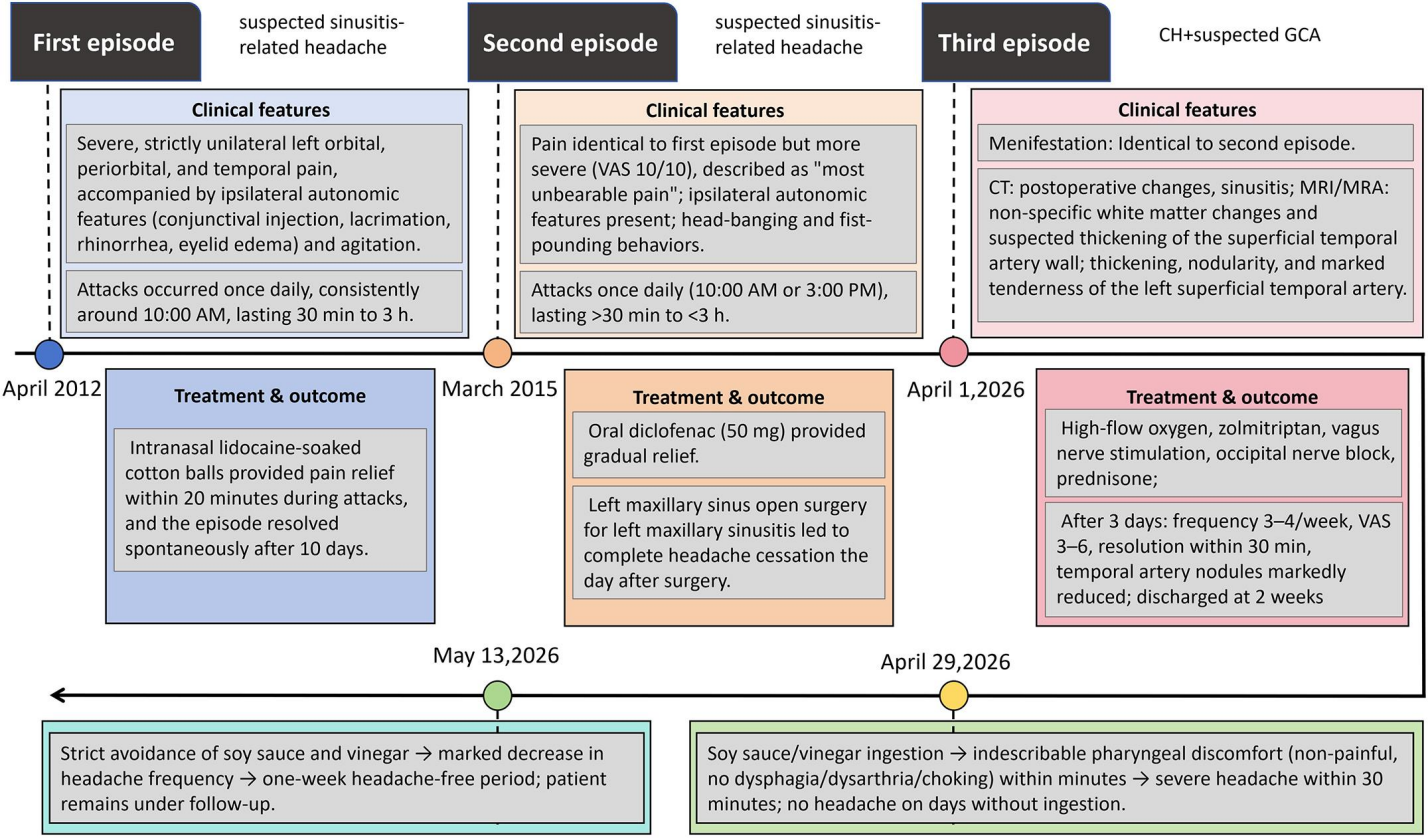
Clinical timeline of the patient' s case. Key events are displayed chronologically, encompassing the evolution of major symptoms and physical signs, serial positive findings from investigations, the diagnostic course, therapeutic interventions, and the patient' s clinical outcome.

At the two-week follow-up, the patient provided crucial information: he recently noted that after consuming soy sauce or vinegar, he would develop an indescribable pharyngeal discomfort (non-painful, without dysphagia, dysarthria, or choking on liquids), invariably followed by severe headache within half an hour. On days when he did not consume soy sauce or vinegar, no headache occurred. Given this presentation, glossopharyngeal neuralgia was considered. High-resolution MRI of the glossopharyngeal nerve was recommended but was declined by the patient. A strict diet avoiding soy sauce and vinegar was advised. At the four-week follow-up, the patient reported that since implementing the dietary elimination, his headache frequency had markedly decreased, and he had been headache-free for one week. He remains under follow-up. Figure 4 illustrates the patient's timeline, depicting the progression of clinical symptoms, diagnosis, and data collected before and after interventions.

## 3 Discussions

This case describes a 53-year-old male with 14 years of episodic CH, complicated by suspected GCA, prior sinusitis-related headache, and novel dietary triggers (soy sauce and vinegar). It highlights diagnostic pitfalls, overlapping phenotypes, and the need for individualized management in complex CH presentations.

The patient's clinical course is complex, and multiple pain-related disorders may have coexisted during the disease process. According to the ICHD-3 criteria, the diagnosis of episodic cluster headache is reasonably certain given the patient's pain characteristics and location (strictly unilateral, left orbital, periorbital, or temporal drilling pain, severe), attack pattern (clustered, periodic), and associated autonomic symptoms (conjunctival injection, lacrimation, rhinorrhea). Atypical temporal artery nodularity and tenderness in the third episode raised suspicion of GCA per 2022 ACR/EULAR criteria (17). The 2022 ACR/EULAR criteria require a mandatory criterion (age ≥50 years at diagnosis) and a cumulative score of ≥6 points. Our patient met the mandatory criterion. In the scoring system, new-onset scalp tenderness (2 points), abnormal temporal artery examination (2 points), and new-onset temporal headache of a type different from previous headaches (possibly 2 points) compel us to consider the possibility of coexistent GCA. However, normal inflammatory markers (ESR, CRP), absence of systemic symptoms, and rapid improvement with CH-directed therapy plus empiric prednisone argued against definite vasculitis, suggesting reactive vascular phenomena rather than true GCA. This overlap underscores the challenge of distinguishing CH from GCA in older adults (6, 7), where scalp tenderness and arterial thickening may mimic vasculitis without histological confirmation.

It must be noted that normal ESR and CRP levels do not definitively exclude the diagnosis of GCA (18). Accumulating evidence indicates that a subset of patients with biopsy-proven GCA can present with normal acute-phase reactants (19). Thus, while the normal inflammatory markers in our patient reduce the pretest probability of GCA, they cannot entirely exclude it, particularly given the concerning physical findings of temporal artery nodularity and tenderness. This is precisely why we initiated empirical glucocorticoid therapy while acknowledging the diagnosis remains unconfirmed.

The coexistence of sinusitis added further complexity to the clinical picture. In the 2015 episode, surgical treatment of left maxillary sinusitis resulted in immediate and complete headache remission. However, upon recurrence in 2026, imaging revealed only postoperative changes and mild mucosal thickening, without evidence of the key link between headache and sinusitis—i.e., obstruction of the drainage pathway (20). These findings appear insufficient to explain the severe, paroxysmal, strictly unilateral phenotype characteristic of cluster headache. Collectively, these unusual observations prompt further exploration of whether sinusitis served as a mimic of cluster headache or rather as a precipitating factor in a predisposed individual. Previous literature (9, 10, 12) can be interpreted as indicating that a potential association between sinusitis and cluster headache may exist, primarily manifesting in two forms: (1) sinusitis acting as a “mimic” of cluster headache, presenting with cluster-headache-like symptoms but essentially representing a secondary headache; and (2) sinusitis serving as a trigger that induces or exacerbates an underlying predisposition to cluster headache. Both sinusitis-related headache and cluster headache share a core pathophysiological mechanism—activation of the trigeminal vascular system—along with a common origin in parasympathetic activation (21). These mechanisms may explain why sinusitis can completely mimic the clinical presentation of cluster headache, rendering the two conditions difficult to distinguish based on symptoms alone.

The diagnostic challenge did not end with sinusitis. During follow-up, the patient repeatedly emphasized that ingestion of soy sauce or vinegar triggered an indescribable pharyngeal discomfort, raising the possibility of glossopharyngeal neuralgia. However, several key features required for this diagnosis were absent. The pain was not described as extremely severe with stabbing, knife-like, electric shock-like, tearing, or burning qualities (22). The typical attack duration of seconds to two minutes was not observed. Moreover, apart from the ingestion of soy sauce or vinegar, other potential triggers such as swallowing, chewing, speaking, coughing, or yawning did not provoke symptoms, and the patient experienced no abnormal sensations when drinking water or eating other foods. Therefore, a definitive diagnosis of glossopharyngeal neuralgia could not be established. In addition, the patient's recurrent unilateral orbital and periorbital pain prompted consideration of headache attributed to trochleitis (23). Nevertheless, the patient did not exhibit the exclusive clinical feature of worsening pain with downward eye movement, which allowed this diagnosis to be reasonably excluded.

Most notably, the patient identified soy sauce and vinegar as potential consistent triggers within the susceptible window period: ingestion preceded throat discomfort and CH attacks within 30 min, with complete remission upon avoidance. Established CH triggers include alcohol, nicotine, and nitrates (16); fermented condiments are scarcely documented. Potential mechanisms involve tyramine, histamine, or osmotic/chemical irritation of the oropharyngeal trigeminal autonomic reflex arc, linking pharyngeal afferents to the trigeminovascular system (1). This finding strongly suggests that certain dietary factors—particularly one or more components common to both condiments—may serve as a definite trigger for cluster headache within the susceptible window period. A compositional analysis of soy sauce and vinegar revealed five shared constituents that have been implicated in headache pathogenesis in the literature: alcohol, histamine, tyramine, monosodium glutamate, and nitrates/nitrites. The academic literature generally identifies alcohol as the strongest and most direct trigger for cluster headache (24). Although only trace amounts of alcohol may remain in soy sauce and vinegar, their vasodilatory and prostaglandin-synthesizing effects should not be underestimated, particularly in sensitive individuals or during vulnerable periods such as the cluster headache window (24). Importantly, the patient in this case never consumes alcohol or any alcohol-containing foods or beverages. Therefore, it is highly plausible that the trace alcohol components present in soy sauce and vinegar are sufficient to trigger his cluster headache attacks. Histamine, through its well-established vasodilatory and pro-inflammatory actions, is a recognized trigger of headaches. Historically, “histaminic cephalalgia” was an early term used to describe CH (25). Although the term “histamine” is no longer used in the disease nomenclature, its role in the pathogenesis of CH—as a precipitating factor during the active cluster period (26) and as a concomitant biochemical alteration during attacks (27)—remains supported by scientific evidence. Both soy sauce and vinegar contain measurable amounts of histamine; notably, red vinegar has been reported to have histamine concentrations as high as 3.66 mg/L. Therefore, the possibility that histamine contributes to this patient's headache attacks cannot be dismissed. Tyramine, a major flavor-forming constituent of both condiments, promotes prostaglandin synthesis and induces vasodilation, and it is well documented in the literature as a headache trigger (28–30); therefore, its potential role is reasonably plausible. Monosodium glutamate (MSG), the primary source of umami taste in soy sauce and vinegar, can trigger acetylcholine release and promote headache sensitization (31), and it is the classic trigger of “Chinese restaurant syndrome” (32). The patient habitually maintains a strict diet and rarely adds MSG to his food; nevertheless, its presence in soy sauce and vinegar means that its potential to trigger headache cannot be excluded. In contrast, nitrates and nitrites, although present in high concentrations in processed meats, are found only in extremely low amounts in soy sauce and vinegar. Moreover, these compounds are ubiquitous in various Chinese foods at certain background levels, making it highly unlikely that they serve as a specific sensitizing trigger for this patient's headache. Based on the above analysis, it remains unclear whether a single shared component is responsible for triggering the patient's attacks or whether multiple components act synergistically. These hypotheses await further validation through controlled provocation studies and larger case series.

This case study has several inherent limitations. First, it is a single-case observational report, which lacks generalizable population-level evidence and cannot establish definitive causal relationships between soy sauce/vinegar intake and cluster headache attacks, only a temporal association. Second, definitive exclusion of GCA was incomplete: the patient declined temporal artery high-resolution MRI and biopsy due to financial constraints, and temporal artery ultrasound was unavailable at our institution; thus, subclinical GCA could not be fully ruled out histologically and radiologically. Third, we failed to conduct standardized dietary provocation testing or blind avoidance challenge to objectively verify the triggering effect of soy sauce and vinegar, relying solely on the patient's self-reported symptom correlation. We acknowledge that the observed temporal relationship between soy sauce/vinegar ingestion and cluster headache attacks, while consistent and remarkable from the patient's perspective, is based solely on self-report and lacks objective verification. In the absence of a structured headache diary, standardized dietary records, or blinded provocation challenges, this association must be regarded as hypothesis-generating rather than confirmatory. No causal inference can be drawn from this single observation. Fourth, the potential underlying mechanism (e.g., histamine, tyramine, trace alcohol, or synergistic effects of multiple ingredients) was only inferred from existing literature, without laboratory detection of biogenic amines or controlled dietary intervention analysis. Fifth, the patient received combined treatment modalities simultaneously, including high-flow oxygen, triptans, non-invasive vagus nerve stimulation, greater occipital nerve block, and empirical prednisone. While this multimodal approach was clinically justified given the severity of the attacks and the concurrent suspicion of GCA, it precludes attribution of the observed clinical improvement to any single intervention. The rapid reduction in attack frequency and pain intensity may reflect a synergistic effect of multiple therapies, the natural resolution of the cluster episode, or a combination of both. We acknowledge this as an inherent limitation of our case report. Sixth, long-term follow-up duration was relatively short, and we could not further observe whether the trigger association remained stable across subsequent cluster periods or whether the headache progressed to chronic cluster headache. Finally, only non-specific white matter changes were observed on brain imaging. No advanced neuroimaging was performed to evaluate the intracranial venous sinuses, hypothalamic region, or cranial nerve vascular pathway abnormalities, thereby limiting further exploration of the patient's unique pathophysiological characteristics.

## 4 Conclusion

This case underscores the diagnostic complexity of CH in middle-aged patients, emphasizing the importance of distinguishing primary CH from GCA, sinusitis-related headache, and other mimicking conditions. It also provides novel clinical evidence that soy sauce and vinegar may act as unique dietary triggers for cluster headache in susceptible individuals. Further large cohort studies, controlled dietary provocation tests, and mechanistic investigations are warranted to validate this trigger relationship, elucidate underlying pathogenic pathways, and optimize individualized preventive and therapeutic strategies for complex cluster headache.

## Data Availability

The original contributions presented in the study are included in the article/Supplementary Material, further inquiries can be directed to the corresponding author.
